# RNA Foci Formation in a Retinal Glial Model for Spinocerebellar Ataxia Type 7

**DOI:** 10.3390/life13010023

**Published:** 2022-12-22

**Authors:** Rocío Suárez-Sánchez, Rodolfo Daniel Ávila-Avilés, J. Manuel Hernández-Hernández, Daniel Sánchez-Celis, Cuauhtli N. Azotla-Vilchis, Enue R. Gómez-Macías, Norberto Leyva-García, Arturo Ortega, Jonathan J. Magaña, Bulmaro Cisneros, Oscar Hernández-Hernández

**Affiliations:** 1 Laboratorio de Medicina Genómica, Departamento de Genética, Instituto Nacional de Rehabilitación-Luis, Guillermo Ibarra Ibarra, Ciudad de México 14389, Mexico; 2 Departamento de Genética y Biología Molecular, Centro de Investigación y de Estudios Avanzados del Instituto Politécnico Nacional, Ciudad de México 07360, Mexico; 3 Departamento de Toxicología, Centro de Investigación y de Estudios Avanzados del, Instituto Politécnico Nacional, Ciudad de México 07360, Mexico; 4 Escuela de Ingeniería y Ciencias, Departamento de Bioingeniería, Tecnológico de Monterrey-Campus Ciudad de México, Ciudad de México 14380, Mexico

**Keywords:** spinocerebellar ataxia type 7, inducible cell models, RNA toxicity, alternative splicing abnormalities

## Abstract

Spinocerebellar ataxia type 7 (SCA7) is a neurodegenerative disorder characterized by cerebellar ataxia and retinopathy. SCA7 is caused by a CAG expansion in the *ATXN7* gene, which results in an extended polyglutamine (polyQ) tract in the encoded protein, the ataxin-7. PolyQ expanded ataxin-7 elicits neurodegeneration in cerebellar Purkinje cells, however, its impact on the SCA7-associated retinopathy remains to be addressed. Since Müller glial cells play an essential role in retinal homeostasis, we generate an inducible model for SCA7, based on the glial Müller MIO-M1 cell line. The SCA7 pathogenesis has been explained by a protein gain-of-function mechanism, however, the contribution of the mutant RNA to the disease cannot be excluded. In this direction, we found nuclear and cytoplasmic foci containing mutant RNA accompanied by subtle alternative splicing defects in MIO-M1 cells. RNA foci were also observed in cells from different lineages, including peripheral mononuclear leukocytes derived from SCA7 patient, suggesting that this molecular mark could be used as a blood biomarker for SCA7. Collectively, our data showed that our glial cell model exhibits the molecular features of SCA7, which makes it a suitable model to study the RNA toxicity mechanisms, as well as to explore therapeutic strategies aiming to alleviate glial dysfunction.

## 1. Introduction

Spinocerebellar ataxia type 7 (SCA7) is an autosomal dominant neurodegenerative disorder characterized mainly by progressive gait ataxia and retinopathy. Common symptoms include dysarthria, dysmetria, hyperreflexia, spasticity, ophthalmoplegia, slow eye movement, and gradual vision loss that eventually leads to blindness [1,2]. Frontal executive dysfunction and sensory-motor peripheral neuropathy have also been reported [3]. SCA7 is caused by an unstable CAG expansion in exon 3 of the *ATXN7* gene, which is mapped on chromosome 3p12-21.1. The CAG repeat is polymorphic; while normal alleles carry up to 35 repeats, the disease-associated alleles contain 36 repeats and over. The expression of expanded CAG repeats results in the translation of mutant ataxin-7 carrying a polyglutamine (polyQ) tract [4,5,6].

Ataxin-7 is a ubiquitous protein that belongs to the highly conserved transcriptional coactivator SPT3-TAF9-ADA-GCN5 acetyltransferase (STAGA) complex; however, the polyQ tract in ataxin-7 disturbs STAGA activity [7,8,9,10]. SCA7 cells frequently contain nuclear aggregates composed of misfolded and/or proteolytic fragments of mutant ataxin-7, as well as proteasome components, chaperons, STAGA subunits, and transcription factors [11,12,13]. The SCA7 mutation triggers numerous deleterious effects, including sustained oxidative stress, activation of pro-apoptotic pathways, mitochondria dysfunction, defective glutamate transport, and excitotoxicity, which collectively leads to neurodegeneration [14,15].

Although mutant ataxin-7 is recognized as the central pathogenic contributor to SCA7, cumulative evidence indicates that expanded CAG repeats may also exert deleterious effects through an RNA toxic gain-of-function mechanism [16,17]. It has been shown that the abnormal binding of proteins to the mutant RNA structures may cause altered cellular functions. For comparison, RNA toxicity has been described for myotonic dystrophy type 1 (DM1), a disease caused by a CTG expansion in the 3′unstranslated region of the *DMPK* gene [18]. In DM1, mutant RNA forms nuclear foci that trigger sequestration/dysfunction of the Muscleblind-like (MBNL) proteins, which act as alternative splicing regulators [19]. Malfunctioning of these proteins yields aberrant splicing of many different genes, contributing to the disease phenotype. A number of neurodegenerative polyglutamine diseases have been described, having in common the CAG tract expansion as the causative disease mutation. They include SCA7, Huntington’s disease (HD), SCA1, SCA2, SCA3, and dentatorubral pallidoluysian atrophy (DRPLA). Interestingly, mutant RNA aggregates have been detected in fibroblasts from patients with these polyQ disorders [20]. In SCA3, the expression of untranslated CAG repeats in *Drosophila* caused the loss of neuronal integrity in the nervous system and the eye [21], while in SCA2, the abnormal interactions between mutant CAG transcript and RNA binding proteins resulted in neurotoxicity and deregulation of ribosomal RNA maturation [22]. Finally, the HD mutant RNA was found to provoke either defective alternative splicing, deregulated microRNA (miRNA) expression, or altered translation [23].

Recent growing evidence indicates that glial dysfunction contributes to the SCA7-associated neuropathology, which takes place in the Purkinje cell layer, the dentate nuclei, and the inferior olivary nuclei of the cerebellum [24,25,26,27,28] as a result of the interaction between Purkinje cells and inferior olive neurons with Bergmann glia. Indeed, a decrease in the glutamate transporter GLAST in Bergmann glia provokes impaired glutamate uptake, which in turn induces excitotoxicity and dark cell death [29,30]. In contrast to the advancement of the role of Bergmann glia in SCA7, the effect of mutant ataxin-7 on Müller glia has been largely overlooked. Although gliosis and progressive activation of Müller cells have been documented, the mechanistic link between glial alterations and photoreceptor cell death is still poorly understood [12,31].

To get initial insight into the potential effects of SCA7 mutation on the Müller retinal cells, we generated a model for SCA7 based on the human Müller MIO-M1 cell line [32]. MIO-M1-Q10 and MIO-M1-Q64 cells express in an inducible manner ataxin-7 carrying a polyQ tract with 10 or 64 residues, respectively. Remarkably, MIO-M1-Q64 cells were able to form protein and RNA aggregates of mutant ataxin-7 in the nucleus, which was accompanied by subtle alternative splicing alterations in MBNL1 splice target genes. The MIO-M1 SCA7 model represents a useful biological tool to study the mechanisms underlying both protein and RNA toxicity in retinal Müller cells.

## 2. Materials and Methods

### 2.1. Plasmid Constructs

A DNA duplex fragment encoding Myc epitope was inserted into the pTRE3G vector, between the SalI and NheI sites, to generate a pTRE3G-Myc plasmid. Then, the PCR-amplified *ATXN7* cDNA carrying 10 CAG repeats was ligated into the NheI site of pTRE3G-Myc to create pTRE3G-Myc-10Q vector. The TRE3G-Myc-64Q vector was engineered by subcloning the full-length *ATXN7* cDNA into the NheI site of the pRSET/EmGFP vector. Then, pRSET/EmGFP-*ATXN7* was digested with AatII/NarI enzymes, and the released fragment was replaced with the AatII/NarI digested fragment obtained from the genomic DNA of a 64 CAG genotyped SCA7 patient. Once replaced, full-length cDNA of mutant *ATXN7* was cloned into the NheI site of the pTRE3G-Myc plasmid to generate pTRE3G-Myc-64Q vector. The transgene insertion and pTRE3G-Myc-10Q and pTRE3G-Myc-64Q vectors’ integrity were confirmed by enzyme digestion and Sanger sequencing. A general workflow of experiments is shown in Figure S1.

### 2.2. Full Length ATXN7 cDNA Amplification

Peripheral blood mononuclear cells were obtained by Lymphoprep density gradient from a healthy subject carrying 10 CAG repeats in the *ATXN7* gene, and total RNA was isolated using TRIzol reagent (Invitrogen, Carlsbad, CA, USA). RNA integrity was evaluated by gel electrophoresis; purity and quantification were determined in a NanoDrop 2000 spectrophotometer (NanoDrop Technologies, Wilmington, DE, USA). Total RNA (1 µg) was retro-transcribed using the high-capacity cDNA reverse transcription kit, according to the manufacturer’s protocol (Thermo Fisher Scientific. Waltham, MA, USA). Then, PCR reactions were performed in 50 µL total volume using the Herculase II Fusion DNA polymerase (Agilent Technologies, Santa Clara, CA, USA). Amplification was performed on a Veriti Thermal Cycler (Applied Biosystems, Foster City, CA, USA) at 96 °C for 3 min as the initial denaturing step, followed by 96 °C for 30 s, 66 °C for 30 s, and 68 °C for 3 min for 30 cycles. The sequence of primers used for *ATXN7* cDNA amplification is shown in Table S1. Genotyping of control and SCA7 subjects was performed by PCR and capillary electrophoresis, as previously described [33].

### 2.3. Cell Culture and Stable Transfection

We used the Tet-On 3G system, in which doxycycline provokes a trans activator to specifically bind to the pTRE3G promoter, activating the transcription of *ATXN7* which is cloned downstream of the promoter in the responding plasmid. MIO-M1 CMV-Tet cells, which constitutively express the trans activator protein [34], were stably co-transfected with the puromycin linear marker (Clontech, Mountain View, CA, USA) and pTRE3G-Myc-10Q or pTRE-3G-Myc-64Q constructs, by using lipofectamine 2000 (Invitrogen, Carlsbad, CA, USA) according to the manufacturer’s instructions. Then, individual puromycin-resistant cells were selected for three weeks. Generated cells were maintained within a humidified 5% CO_2_ atmosphere at 37 °C in Dulbecco’s Modified Eagle Medium (DMEM) (Invitrogen, Carlsbad, CA, USA), supplemented with 10% fetal bovine serum (FBS), 100 U/mL penicillin, 100 µg/mL streptomycin, 350 µg/mL geneticin (G418), and 0.35 µg/mL puromycin. Subsequent induction experiments were performed with 1 µg/mL doxycycline (or different concentration when indicated) in culture media containing 10% FBS tetracycline-free. For all experiments, the induction medium was changed every third day.

### 2.4. Western Blotting Analysis

Whole-cell lysates were obtained from confluent cells grown in 60 mm culture plates. Briefly, cells were washed with cold phosphate-buffered saline pH 7.4 (PBS: 137 mM NaCl, 2.7 mM KCl, 10.1 mM Na_2_HPO_4_, 1.8 mM KH_2_PO_4_) and lysed on ice during 20 min in triple detergent lysis buffer (50 mM Tris-Cl pH 8.0, 150 mM NaCl, 0.1% sodium dodecyl sulfate, 1.0% nonidet P-40, 0.5% sodium deoxycholate, 1 mM phenylmethylsulphonyl fluoride, and 1X complete protease inhibitor cocktail (Roche Applied Science, Penzberg, Germany)). Protein extracts were clarified by centrifugation at 7500× *g* for 10 min at 4 °C, and protein concentrations were determined using the Bio-Rad DC protein assay (Bio-Rad, Hercules, CA, USA). Protein aliquots (70 µg) were mixed with Laemmli buffer, boiled for 5 min, and loaded onto 7.5% SDS-polyacrylamide gels for electrophoresis. Resolved proteins were transferred onto a PVDF membrane using a transblot apparatus (Bio-Rad, Hercules, CA, USA), blocked with 6% non-fat milk in TBS-T for 1 h, and incubated overnight with anti-ataxin-7 antibody (ab95013) or anti-Myc epitope antibody (sc-40). After three washes in TBS-T (10mMTris-HCl Ph 8.0, 150 mM NaCl, 0.05% (*v*/*v*) Tween-20), membranes were incubated with the corresponding horseradish peroxidase (HRP)-conjugated secondary antibodies (Abcam, Cambridge, UK) and developed using the Western Lightning Plus-ECL system (PerkinElmer, Waltham, MA, USA). Loading control signal was obtained by incubation with an anti-actin antibody (sc-376421) after membrane stripping with 0.2 M NaOH. Protein bands were visualized using the ChemiDoc system, and densitometric analysis was performed with the Image Lab software v 6.1.0 (Bio-Rad, Hercules, CA, USA).

### 2.5. Indirect Immunofluorescence

Cells seeded on glass coverslips were washed with PBS, fixed with 4% paraformaldehyde for 10 min, permeabilized 5 min with 0.2% triton X-100 in PBS, and blocked by exposure to gelatin 0.5% and 1.5% FBS for 20 min at room temperature. Primary anti-ataxin-7 (ab11434) and anti-Myc (sc-40) antibodies were incubated overnight at 4 °C. Cells were washed and then incubated with the goat anti-rabbit IgG antibody (H + L) fluorescein (FI-1000) and horse anti-mouse IgG antibody (H + L) DyLight 594 (DI-2594) secondary antibodies (Vector Laboratories Inc., Burlingame, CA, USA). After washing in PBS, coverslips were mounted on microscope slides with VectaShield antifade medium containing diamino-2-phenylindole (DAPI) (Vector Labs Inc., Burlingame, CA, USA), and examined on a confocal laser scanning microscope (TCP-SP5, Leica, Heidelberg, Germany).

### 2.6. RT-PCR and Alternative Splicing Evaluation

TRIzol reagent (Invitrogen, Carlsbad, CA, USA) was used to isolate total RNA from cell cultures. RNA purity, integrity, and quantification were evaluated as described above. The high-capacity cDNA reverse transcription kit (Thermo Fisher Scientific. Waltham, MA, USA) was used to prepare cDNA from 1 μg total RNA according to the manufacturer’s protocol. PCR reactions were performed in 12.5 μL total volume using the Platinum Taq DNA polymerase (Invitrogen, Carlsbad, CA, USA). Oligonucleotide primer sequences used to determine *ATXN7* and TATA-Binding protein (*TBP)* expression and alternative splicing patterns of *MBNL1, MBNL2,* amyloid beta precursor protein *(APP),* and microtubule-associated protein tau (*MAPT)* are shown in Table S1. The percentage of exon inclusion (PSI) was calculated as (exon inclusion band/(exon inclusion band+ exon exclusion band)) × 100 [35].

### 2.7. TaqMan Assay

The expression of the *ATXN7* transcript was evaluated by retro transcription and quantitative PCR (RT-qPCR) by a TaqMan assay (Applied Biosystems, Foster City, CA, USA). In this assay, the forward primer recognized part of the Myc epitope sequence within the plasmid constructs, thus being specific to exogenous *ATXN7.* PCR reactions were performed in 20 μL total volume reactions containing 100 ng cDNA, 2X TaqMan Universal Master Mix II with UNG (Applied Biosystems, Foster City, CA, USA), 1 μL of TaqMan probe, and 1 μL of primer limited probe for the endogenous control glyceraldehyde-3-phosphate dehydrogenase (*GAPDH*). Amplification reactions were performed on a StepOne Real-Time PCR System (Applied Byosistems, Foster City, CA, USA). Amplification parameters were an initial step of 50 °C for 2 min and 95 °C for 10 min followed by 40 cycles of 95 °C for 15 s, 60 °C for 1 min. Exogenous *ATXN7* relative expression was calculated by using the 2^ΔΔCT^ method.

### 2.8. RNA Fluorescence in Situ Hybridization (RNA-FISH)

Cells cultured on coverslips were fixed with 4% paraformaldehyde for 10 min at room temperature, permeabilized with cold 2% acetone for 5 min, and then incubated overnight in 70% ethanol. Prehybridization was performed in 30% formamide, 2X SSC buffer for 10 min at room temperature, and further incubation for 3 h in a humidified chamber at 37 °C with hybridization buffer [2X SSC, 40% formamide, 0.02% BSA, 2mM vanadyl ribonucleoside (Sigma-Aldrich, St. Louis, MO, USA), 66 μg/mL yeast tRNA (Sigma-Aldrich, St. Louis, MO, USA), and 2 nM TYE563-conjugated LNA (CTG)_6_ probe [20]. Cells were washed in prehybridization buffer for 30 min at 45 °C, soaked in 1X SSC at room temperature and then in PBS. Coverslips were mounted on microscope slides with VectaShield containing DAPI (Vector Labs Inc., Burlingame, CA, USA), and images were captured on a confocal laser scanning microscope (TCP-SP5, Leica, Heidelberg, Germany).

### 2.9. RNA-FISH Coupled to Immunofluorescence

After the post-hybridization wash step of the RNA-FISH protocol, cells were incubated in 3% BSA for 15 min and then incubated overnight at 4 °C with anti-MBNL1 (ab45899) or MBNL2 (ab171551) primary antibodies (Abcam, Cambridge, UK). After a wash in PBS, cells were incubated at room temperature for 1 h with the fluorescein-conjugated anti-rabbit antibody (Vector Labs., Burlingame, CA, USA). Coverslips were mounted with Vectashield Antifade Mounting Medium with DAPI (Vector Labs., Burlingame, CA, USA), and analyzed on a confocal laser scanning microscope (TCP-SP5, Leica, Heidelberg, Germany).

### 2.10. Transient Transfection

SH-SY5Y, N1E-115, C2C12, and HeLa cells seeded on coverslips were transiently co-transfected with pCMV-Tet3G and pTRE3G-Myc-10Q or pTRE3G-Myc-64Q plasmids by using lipofectamine 2000 (Invitrogen, Carlsbad, CA, USA) according to manufacturer’s instructions. At 24 h post-transfection, cells were induced with doxycycline (1 μg/mL) for 24 h, then washed and fixed with 4% PFA prior to be subjected to RNA-FISH analysis.

### 2.11. Statistical Analysis

To determine statistical significance, a two-tailed Student’s t test was used when two groups were compared. When more than two groups were compared, a one-way ANOVA analysis was performed. GraphPad Prism 9.4.1 was used for calculations. Data are expressed as mean ± standard error of the means (±SEM). A significant level was set at *p* < 0.05.

## 3. Results

### 3.1. Generation of a Retinal Glial Cell-Based Model for SCA7

**Figure 1:**
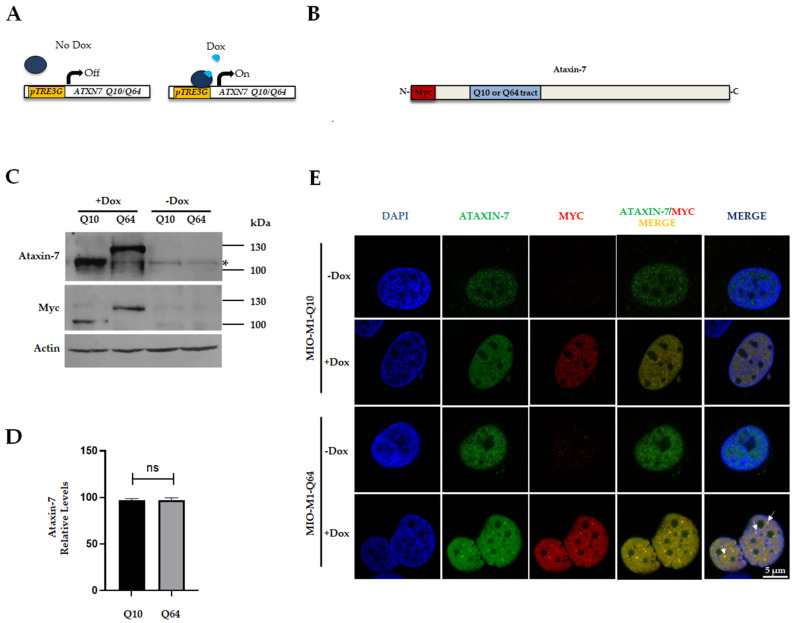
Inducible expression of mutant ataxin-7 elicits nuclear protein aggregates in MIO-M1-Q64 cells: (**A**) Schematic representation of Dox-induced *ATXN7* transgene expression. Transactivator protein binds pTRE3G promoter only in the presence of Dox, which induce the expression of *ATXN7*. (**B**) Schematic representation of N-terminal Myc epitope-tagged human ataxin-7 harboring either 10 or 64 glutamines. (**C**) MIO-M1-Q10 and MIO-M1-Q64 cells were cultured for three days with (+Dox) or without (−Dox) doxycycline. Cell lysates were analyzed by Western blotting using antibodies against ataxin-7, myc epitope and actin (loading control). The asterisk indicates the predicted position of the endogenous ataxin-7. (**D**) The ataxin-7 levels were measured from three independent replicates, with no statistically significant differences between MIO-M1-Q10 and MIO-M1-Q64 cells. The graph represents the relative ataxin-7 expression corrected for actin loading control, ns: not significant (**E**) MIO-M1-Q10 and MIO-M1-Q64 cells, cultured as per (**C**), were immunostained for ataxin-7 using anti-ataxin-7 and anti-myc epitope antibodies, and counterstained with DAPI to visualize nuclei, prior to be imaged by CLSM. Typical optical Z-sections are shown. Mutant ataxin-7 aggregates are denoted by arrows.

We establish a retinal glial cell model of SCA7 using MIO-M1 CMV-Tet cells, a Müller glia cell line that constitutively expresses the Tet-On 3G trans activator protein [34]. MIO-M1 CMV-Tet cells were stably transfected with the appropriate vectors to express in an inducible manner c-myc-tagged-human ataxin-7 proteins harboring 10 (MIO-M1-Q10) or 64 (MIO-M1-Q64) glutamine residues when cultured in the presence of doxycycline (Figure 1A,B). The expression of exogenous ataxin-7 (Q10) or polyQ-expanded ataxin-7 (Q64) (~100 and ~130 kDa, respectively) were observed after 3 days of doxycycline (Dox) induction, as shown by Western blot analysis using antibodies against ataxin-7 and c-myc, while endogenous ataxin-7 was detected using ataxin-7 antibody (Figure 1C). The protein level of exogenous ataxin-7 was similar between MIO-M1-Q10 and MIO-M1-Q64 cells (Figure 1D).

Since nuclear inclusions of mutant proteins are hallmarks of polyQ diseases, we were prompted to ascertain whether polyQ-expanded ataxin-7 (Q64) formed nuclear foci using c-myc antibody and confocal laser scanning microscopy (CLSM) analysis. A predominant nuclear localization of both ataxin-7 Q10 and ataxin-7 Q64 was found upon Dox induction; however, solely ataxin-7 Q64 had the ability to form nuclear foci (Figure 1E). Endogenous ataxin-7 also accumulated in the nucleus, as shown by immunolabeling-uninduced MIOM1-Q10 and MIO-M1-Q64 cells with ataxin-7 antibody. Overall, these results show the feasibility of the cell model to analyze, in a controlled manner, the effect of the polyQ-expanded ataxin-7 on Müller glial cell physiology.

### 3.2. MIO-M1-Q64 Cells Contain Ataxin-7 Ribonuclear Foci

**Figure 2:**
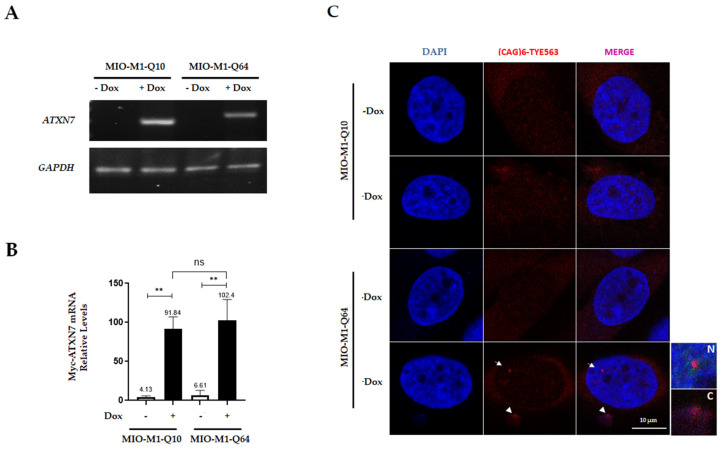
Mutant ATXN7 expression triggers RNA foci formation in MIO-M1-Q64 cells. (**A**) Expression of ATXN7 transgene was analyzed by RT-PCR in MIO-M1-Q10 and MIO-M1-Q64 cells before (−Dox) and after (+Dox) doxycycline treatment (1 µg/mL) for three days. GAPDH was used as endogenous control. (**B**) The inducible expression of exogenous ATXN7 evaluated by RT-qPCR in MIO-M1-Q10 and MIO-M1-Q64 cells before (−Dox) and after (+Dox) doxycycline induction (1µg/mL) for three days, using GAPDH as endogenous control. Data correspond to the mean ±SEM from three independent experiments, with significant differences determined by a one-way ANOVA analysis, **: *p* < 0.005, ns: no significance. (**C**) Ataxin-7 RNA foci were visualized by RNA-FISH using a TYE563-conjugated LNA (CTG)_6_ probe. Cells cultured on coverslips were subjected to Dox treatment (+Dox) for 3 days to induce exogenous ataxin-7 expression. Nuclei were visualized by DAPI staining prior to CLSM analysis. Representative single optical Z-sections are shown and the presence of nuclear and cytoplasmic foci is denoted with white arrow and head arrow, respectively. The white square inserts indicate the magnified area of nuclear (N) and cytoplasmic (C) RNA foci.

**Figure 3:**
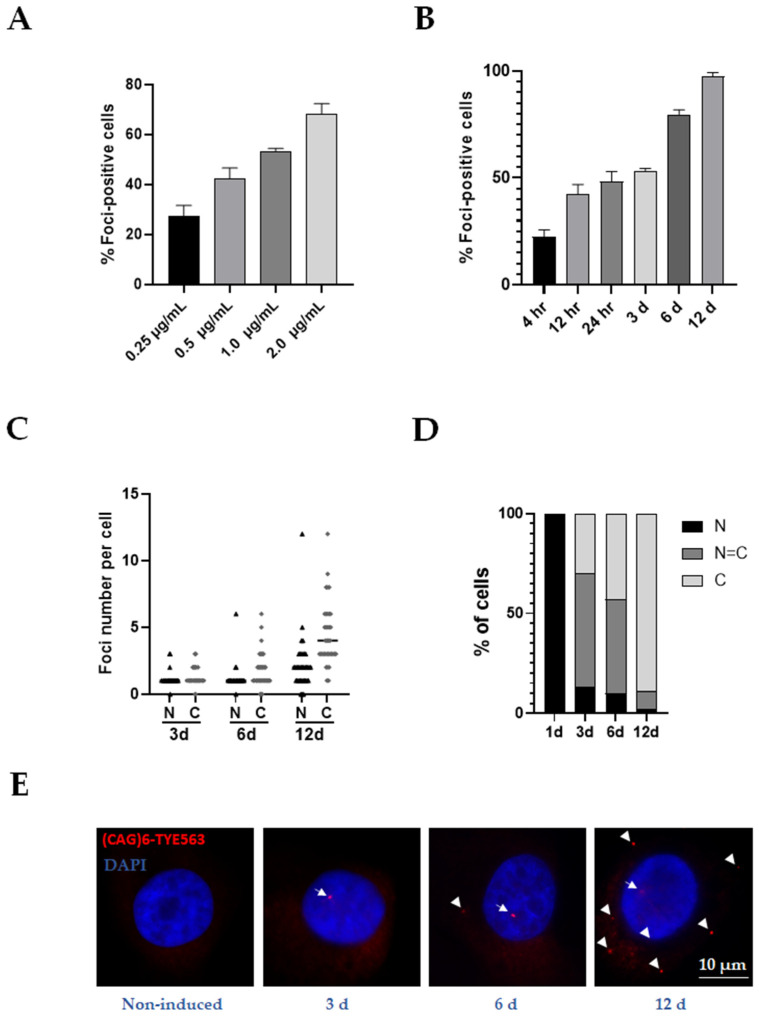
Mutant RNA foci inducibility in MIO-M1-Q64 cells. (**A**) A dose-response curve was obtained by RNA-FISH using different concentrations of doxycycline in MIO-M1-Q64 cultures evaluated at three days of induction. Data shown are means ± SEM from at least three independent experiments. n = 80 cells/group (**B**) Percentage of foci-positive cells over induction time. RNA-FISH was performed on MIO-M1-Q64 cells at the indicated time points during doxycycline induction (1 µg/mL). Data shown are means ± SEM from three independent experiments. n = 80 cells/group. (**C**) Count nuclear/cytoplasmic foci in MIO-M1-Q64 cells at the indicated induction days. n = 60 cells/group from three independent experiments. (**D**) Subcellular localization of foci in MIO-M1-Q64 cells induced at the indicated induction days. (**E**) Representative FISH-RNA micrographs of MIO-M1-Q64 cell cultures at the indicated days of doxycycline induction (1 µg/mL). Data shown are means ± SEM from at least three independent experiments. n = 80 cells/condition. White arrows and head arrows denote nuclear foci and cytoplasmic foci, respectively.

Previous evidence shows that expanded CAG repeats can exert their pathogenic effects through an RNA toxicity mechanism [16,17]. Thus, we explored the possibility that mutant ataxin-7 transcript is assembled into nuclear RNA foci in MIO-M1-Q64 cells. Firstly, the expression of exogenous ataxin-7 mRNA was analyzed by retro-transcription and end point PCR (RT-PCR) experiments, using a c-myc epitope-specific forward primer. Ataxin-7 transcripts were detected in both MIO-M1-Q10 and MIO-M1-Q64 cells, specifically after doxycycline induction (3 days). In contrast, the ataxin-7 mRNAs were not detected in MIO-M1-Q10 and MIO-M1-Q64 cells cultured without doxycycline (Figure 2A). Subsequent real-time PCR (RT-qPCR) assays confirmed the specificity and inducibility of the Tet-On 3G system. Virtually no expression was detected in uninduced cells cultures, while Dox-induced cells exhibited a robust increase of ataxin-7 mRNA in both MIO-M1-Q10 (22.3-Fold Change, *p* = 0.0047) and MIO-M1-Q64 (15.5-Fold Change, *p* = 0.0087) cells (Figure 2B). It is worth mentioning that exogenous ataxin-7 mRNA was expressed at a comparable level between MIO-M1-Q64 cells and MIO-M1-Q10 cells before (*p* = 0.7745) and after doxycycline induction (*p* = 0.7884) (Figure 2B). Next, RNA-FISH assays were carried out to decorate ataxin-7 RNA foci using a (CTG)_6_-TYE563 probe. Remarkably, small nuclear and cytoplasmic RNA foci were found specifically in Dox-induced MIO-M1-Q64 cells (Figure 2C), suggesting the involvement of mutant ataxin-7 transcripts in SCA7 pathophysiology in retinal glial cells. In order to enhance RNA foci formation, experimental conditions for ataxin-7 induction were optimized. MIO-M1-Q64 cells were treated with Dox over a range of concentrations (from 0.25 µg/mL to 2 µg/mL) and times (from 4 h to 6 d). The higher the concentration of Dox, the higher the number of foci-positive cells (Figure 3A). As high Dox dose provokes undesired effects on cell physiology [36], 1 µg/mL Dox concentration was chosen for further experiments. Similarly, the longer the Dox treatment, the higher the number of foci-positive cells (Figure 3B). Interestingly, although the number of both the nuclear and the cytoplasmic RNA foci per cell increased in direct proportion to the Dox induction time, their predominant localization shifted from the nucleus to the cytoplasm (Figure 3C–E).

### 3.3. MIO-M1-Q64 Cells Display Subtle Alternative Splicing Abnormalities

**Figure 4:**
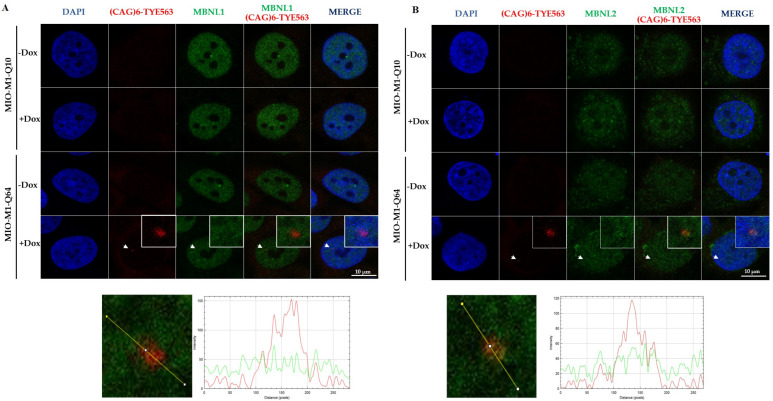
Suble co-localization between MBNL splicing factors and nuclear RNA aggregates. (**A**) RNA-FISH [(TYE563-(CTG)_6_ probe] and immunofluorescence (MBNL1) showed mild co-localization of mutant ATXN7 RNA with MBNL1 in the nucleus of MIO-M1-Q64 induced cells at three days of doxycycline induction. Lower panel shows the plot profile of the section shown as a line. Fluorescence-intensity distribution for MBNL1 and TYE563-(CTG)_6_ probe are indicated in green and red, respectively. (**B**) RNA-FISH coupled to immunofluorescence for MBNL2. Cells were counterstained with DAPI prior to being analyzed by confocal microscopy. Lower panel shows the plot profile of the section shown as a line. Fluorescence-intensity distributions for MBNL2 and TYE563-(CTG)_6_ probe are indicated in green and red respectively. Representative single typical optical Z-sections are shown. The white square inserts indicate the magnified area of nuclear foci (white arrows).

As CAG-containing mutant transcripts are retained in the nucleus within splicing bodies in different polyQ diseases, compromising the alternative splicing mechanism [17], we were prompted to ascertain whether mutant ATXN7 mRNA adversely influences this cellular process. Specifically, alternative splicing defects may result from sequestration of MBNL splicing factors by ribonuclear aggregates. Thus, RT-PCR experiments on MIO-M1-Q10 and MIO-M1-Q64 cells were carried out to analyze the alternative splicing regulation by using primers flanking regulated exons of the selected genes (Table S1). Our data revealed a statistically significant increase in the percentage of splicing inclusion (PSI) for MBNL1 exon 7 in MIO-M1-Q64 induced cells when compared to MIO-M1-Q64 non-induced cells (mean PSI 25.77 vs. 23.40, *p* = 0.0185), MIO-M1-Q10 doxycycline-treated cells (mean PSI 25.77 vs. 23.03, *p* = 0.0428), and MIO-M1-Q10 non-induced cells (mean PSI 25.77 vs. 22.08, *p* = 0.0015) (Figure S1). Unexpectedly, we did not observe any changes in PSI for MBNL2 exon 7, APP exon 8, and MAPT exon 10 (Figure S1). In agreement with this, FISH-RNA coupled to immunofluorescence experiments reveal mild co-localization of nuclear RNA foci with MBNL1 and MBNL2 in MIO-M1-Q64 cells induced for 3 days, as revealed by CLSM and the line profile of fluorescence-intensity distribution (Figure 4).

**Figure 5:**
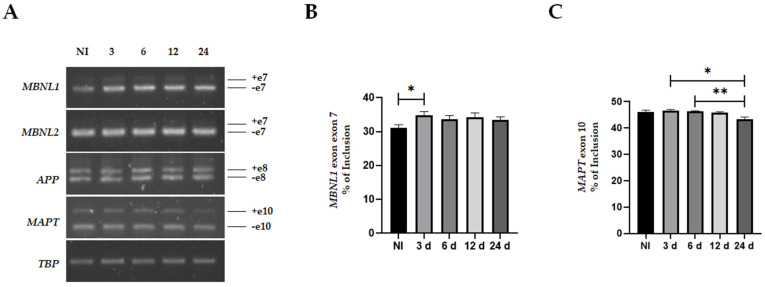
Subtle alternative splicing defects in MIO-M1-Q64 cells. (**A**) Alternative splicing evaluation was carried out at the indicated days of induction (3–24 days) by RT-PCR in MIO-M1-Q64 cells cultured in the presence of 1 µg/mL doxycycline for 24 days. NI: Non-induced control. Representative images of three independent experiments are shown. TBP expression was used as endogenous control. (**B**) The percentage of splicing inclusion of MBNL1 exon 7 was calculated. Data shown are means ± SEM of independent experiments, with significant differences determined by a one-way ANOVA analysis. *: *p* < 0.05. (**C**) The percentage of splicing inclusion of MAPT exon 10 was calculated. Data shown are means ± SEM of independent experiments, with significant differences determined by a one-way ANOVA analysis. *: *p* < 0.05; **: *p* < 0.005. NI: Non-induced; 3d–24d: days of induction.

Subsequently, we performed alternative splicing evaluation in MIO-M1-Q64 cells under the extended induction scheme. Surprisingly, we did not notice changes in the MBNL2 exon 7 or APP exon 8 with this long induction (Figure 5A). However, we confirmed the observed alteration in MBNL1 exon 7 when comparing non-induced cells with induced cells for 3 days (31.06 PSI vs. 34.86 PSI *p* = 0.0472). Intriguingly, this effect did not accentuate over time (Figure 5A,B). In these experiments, we also detected a significant decrease in MAPT exon 10 PSI on day 24 compared to 3 and 6 days of induction (43.41 PSI vs. 46.55 PSI *p* = 0.0479; and 43.41 PSI vs. 46.22 PSI *p* = 0.0046) (Figure 5A,C).

**Figure 6:**
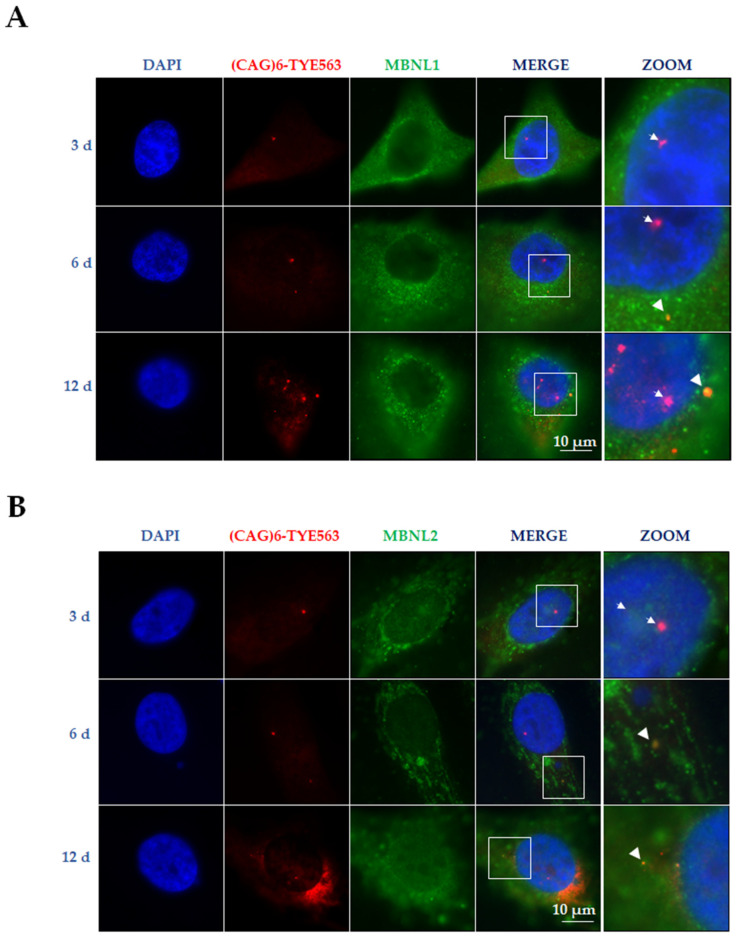
MBNL splicing factors co-localize with cytoplasmic RNA foci in MIO-M1-Q64 cells. (**A**) RNA-FISH [(TYE563-(CTG)_6_ probe] and immunofluorescence (MBNL1) experiments showed co-localization of mutant ATXN7 RNA with MBNL1 in the cytoplasm of MIO-M1-Q64 (**B**) RNA-FISH coupled to immunofluorescence for MBNL2. Cells were counterstained with DAPI prior to being analyzed by confocal microscopy. Note that co-localization of MBNL1/2 was more distinctive with cytoplasmic RNA foci (head arrows) compared to nuclear RNA foci (white arrows) mainly at 6 (6 d) and 12 (6 d) days of induction. The white square inserts indicate the magnified area.

To assess whether MBNL splicing factors co-localize with RNA foci in an extended doxycycline treatment, we carried out RNA FISH and immunostaining experiments in MIO-M1-Q64 cells induced over 12 days. Outstandingly, we observed co-localization between cytoplasmic RNA aggregates with both MBNL1 and MBNL2, which implies that other MBNL functions, rather than splicing, could be affected (Figure 6A,B).

### 3.4. Induction of Ataxin-7 RNA Foci in Cell Lines of Different Lineages

**Figure 7:**
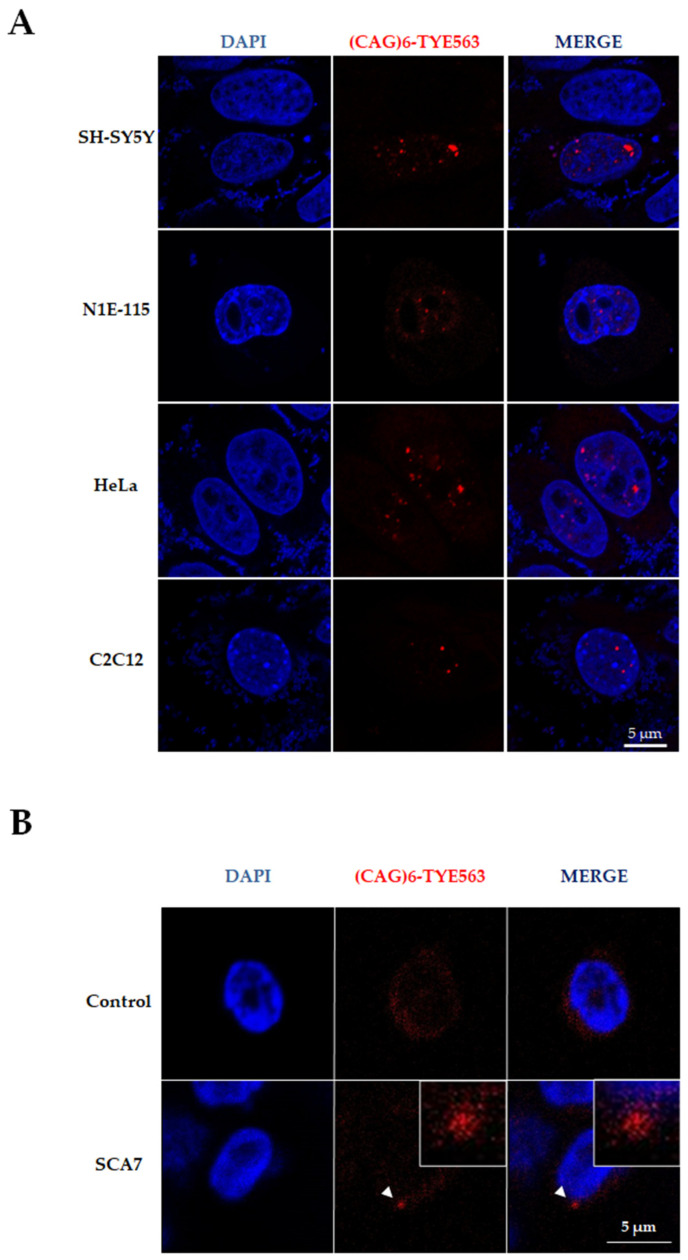
Mutant RNA foci formation is a cell-type-independent phenomenon in SCA7. (**A**) RNA-FISH showed RNA formation in several cell lines transiently co-transfected with pCMV-Tet3G and pTRE3G-Myc-64Q plasmids. Cells were induced 24 h post-transfection. After RNA-FISH, cells were counterstained with DAPI prior to being analyzed by confocal microscopy. Representative single typical optical Z-sections are shown. (**B**) RNA-FISH revealed RNA foci in peripheral mononuclear cells of SCA7 patients. The white square insert indicates the magnified area of RNA foci (head arrow).

To understand whether mutant RNA foci formation depends on the cell type or is a generalized cell type-independent phenomenon, we executed transient co-transfection assays of pCMV-Tet3G (trans activator) and pTRE3G-Myc-64Q plasmids on cells from different lineages. We revealed the presence of RNA foci in SH-SY5Y and N1E-115 neuroblastoma cell lines, HeLa epithelial cells, and the C2C12 myoblast cell line after doxycycline induction (Figure 7A). Remarkably, we also observed RNA aggregates in peripheral mononuclear cells from SCA7 patients carrying a (CAG)_53–62_ expansion, (Figure 7B), suggesting that RNA foci are a widespread process in SCA7.

## 4. Discussion

In this work, we reported the generation of a MIO-M1 cell-based glial model for SCA7, which expresses ataxin-7 (MIO-M1-Q10 cells) or polyQ-expanded ataxin-7 (MIO-M1-Q64 cells) under the control of the Tet-On 3G system. The MIO-M1 cell line has been used to study the role of Müller cells under normal and pathological conditions [37,38,39] because they maintain functional features of Müller cells, including the response to glutamate, the expression of the cell markers (CRALB, EGF-R and glutamate synthetase), and the presence of progenitors’ characteristics [32,40].

A similar expression of the exogenous ataxin-7 was observed between MIO-M1-Q10 and MIO-M1-Q64 cells upon doxycycline induction. Consistently, the level of the mutant *ATXN7* transcript was not different from the wild type in the total brain of the Sca7^266Q/5Q^ mouse model [12]. Contrastingly, increased *ATXN7* transcript levels were observed in SCA7 human fibroblasts as well as in the cerebellum and retina of SCA7 mice models [41]. It has been proposed that SCA7 mutation affects the SAGA complex transcriptional activity, causing in turn a downregulation of miR-124 (a negative regulator of *ATXN7*), which ultimately might induce increased *ATXN7* expression [41]. Further studies are required to analyze whether miR-124 regulates the ataxin-7 transcript levels in MIO-M1-Q64 cells.

Previously, the inducible expression of exogenous poly-Q expanded ataxin-7 has been employed to unveil the mechanisms underlying SCA7 in neuronal cells. The use of a stable inducible PC12 cell model expressing polyQ expanded ataxin-7 has revealed alterations in the RNA regulatory function of FUS [42] as well as impaired function of p53 and NOX1 [43]. Interestingly, the expression of the mutant ataxin-7 elicited the formation of protein aggregates in MIO-M1-Q64 cells. Protein inclusions are a hallmark of polyQ diseases [44,45,46,47], and SCA7 is not an exception. Recent studies demonstrated the formation of nuclear protein inclusions of mutant ataxin-7 in Bergmann glial cells of the cerebellum, and primary cultured astrocytes [29,48]. Protein aggregates appear to contribute to the SCA7 neuropathophysiology, as evidenced by the fact that mice expressing mutant ataxin-7 specifically in Bergmann glia lose Purkinje neurons and exhibit symptoms of disease onset [27]. Future experiments are needed to assess the adverse effects of protein aggregates on the physiology of MIO-M1-Q64 glial cells.

An exciting result was the detection of nuclear and cytoplasmic RNA foci in MIO-M1-Q64 cells. The number of ataxin-7 RNA foci per cell increased in a way that depends on both Dox concentration and the exposure time to the inductor. Thereby, it is plausible to hypothesize that the number and size of RNA foci will increase with the number of CAG repeats within the *ATXN7* gene, in a similar way to that which occurs in HD and SCA3 fibroblasts, where the foci number is positively correlated with the CAG repeat length [17]. RNA foci are considered a molecular signature of RNA toxicity because of the ability of mutant CAG transcripts to aggregate in fibroblasts, lymphoblasts, iPS cells, and neuronal progenitors from polyQ diseases, including HD, SCA3, and DRLPA [17].

Extensive evidence obtained from the study of DM1, the prototype of a RNA toxicity disease, indicates that RNA foci interfere with the function of MBNL splicing factors by sequestering them into nuclear foci [19,49,50]. Unlike what was observed in DM1, we found subtle alternative splicing defects in MIO-M1-Q64 cells, namely deregulation in *MBNL1* exon 7 inclusion and *MAPT* exon 10 inclusion. The observation that nuclear foci colocalized at some extent with MBNL factors might be functionally linked to the subtle alternative splicing defects observed in MBNL1 splice target genes. Several functional consequences of *MBNL1* and *MAPT* mis-splicing in MIO-M1 cells can be anticipated. Recently, MBNL-dependent splicing defects affecting mRNAs that control cell adhesion and spreading have been reported in DM1 astrocytes [51]. It has been described that *MBNL1* exon 7 is necessary for MBNL dimerization and regulation of mRNAs involved in cell migration and DNA repair [52]. In addition, it has been shown that *MBNL1* exon 7 enhances the sequestration of MBNLs in nuclei of DM1 cells and thus contributes to the severity of the phenotype by promoting MBNLs interactions [53]. Activated microglia, gliosis, and neuroinflammation are hallmarks of Tau pathology and neurodegeneration [54]. Interestingly, a preferential expression of *MAPT* exon 10, promoted by the STOX1A transcription factor which is involved in late-onset Alzheimer’s disease, is observed in glial cell cultures [55]. In addition, it has been described that *MAPT* exon 10 improves the ability of Tau to bound microtubules and favor their polymerization [56,57], thus *MAPT* isoform-lacking exon 10 would interfere in MIO-M1 SCA7 model with microtubules polymerization affecting the cytoskeleton dynamics. Interestingly, augmentation of tau exon 10 inclusion was reported in the cortex and putamen of HD samples [58], suggesting that this splicing event may be relevant in the context of polyQ diseases. Furthermore, abnormal splicing at exon 10 is sufficient to cause neurodegeneration [59,60]. Thus, the deep evaluation of the functional consequences of *MBNL1* exon 7 and *MAPT* exon 10 splicing alterations in MIO-M1-Q64 cells is deserved. RNA-mediated toxicity may arise from aberrant interactions between mutant RNA and its protein partners in specific cell compartments. Several studies have demonstrated that mutant CAG mRNAs sequester proteins, including MBNL1, SRSF6, U2AF65, and nucleolin, alter key cellular mechanisms including alternative splicing regulation, ribosomal RNA maturation, recruitment of translation factors, and deregulation of the microRNA machinery [22,61,62,63]. Undoubtedly, any alteration in the aforementioned processes could impact on Müller cells’ function. In the retina, alternative splicing deserves special attention: the retina expresses tissue-specific splicing factors and exclusive tissue-specific exons [64]. The occurrence of RNA foci in MIO-M1-Q64 cells open new avenues into the study of alternative splicing of retinal genes.

Noteworthily, the ataxin-7 cytoplasmic RNA foci co-localized to a certain extent with MBNL1 and MBNL2 proteins in MIO-M1-Q64 cells. The MBNL protein family is involved in multiple cellular processes, such as alternative splicing, alternative polyadenylation, mRNA nuclear export, miRNA processing, and translation regulation [65,66]. Specifically, it has been reported that cytoplasmic MBNL1 promotes mRNA stability and neuron outgrowth [67,68,69]. Therefore, our results imply that MBNL sequestration by cytoplasmic foci might contribute, at least in part, to the molecular alterations observed in SCA7. Identification of the RNA foci protein components is required to gain insight into the mechanisms underlying RNA toxicity in SCA7.

In this study, the exogenous expression of ataxin-7 generates RNA foci in several cellular lines, including neuronal, epithelial, and muscular cells, which highlights the feasibility of our inducible system to analyze the SCA7-associated RNA toxicity in different cellular environments. Finally, the observation that RNA foci occur in peripheral mononuclear leukocytes derived from SCA7 patients, an easily accessible tissue, opens new avenues toward the use of this molecular signature as a biomarker for disease diagnosis and/or progression.

## 5. Conclusions

We have developed an inducible cell model for SCA7 based on the glial retinal MIO-M1 cells which recreate the molecular signatures of the disease, including the presence of ataxin-7 protein inclusions and RNA foci associated with mild alternative splicing abnormalities. MIO-M1-Q64 cells will enable us to study the ataxin-7 RNA-mediated toxicity in glial cells, as well as to explore therapeutic strategies against SCA7.

## Data Availability

Not applicable.

## Supplementary Materials

The following supporting information can be downloaded at: https://www.mdpi.com/article/10.3390/life13010023/s1, Table S1: List of primers used in this study. Figure S1: General workflow. Figure S2: Alternative splicing of exon 7 in MBNL1 is dysregulated in MIO-M1-Q64 cells.
