# Supplementary material for: RNA Foci Formation in a Retinal Glial Model for Spinocerebellar Ataxia Type 7
Source: Life (Basel). 2022 Dec 22;13(1):23. doi: 10.3390/life13010023 (PMC9861853; doi:10.3390/life13010023)
Supplement: Supplementary file 1 [file life-13-00023-s001.zip › life-2083103-supplementary.pdf]

life

Article

RNA Foci Formation in a Retinal Glial Model for Spinocerebellar Ataxia Type 7

Rocío Suárez-Sánchez [1], Rodolfo Daniel Ávila-Avilés [2], J. Manuel Hernández-Hernández [2], Daniel Sánchez-Celis [2], Cuauhtli N. Azotla-Vilchis [2], Enue R. Gómez-Macías [2], Norberto Leyva-García [1], Arturo Ortega [3], Jonathan J. Magaña [1,4], Bulmaro Cisneros [2] and Oscar Hernández-Hernández [1,*]

1 Laboratorio de Medicina Genómica, Departamento de Genética, Instituto Nacional de Rehabilitación-Luis, Guillermo Ibarra Ibarra, Ciudad de México 14389, México
2 Departamento de Genética y Biología Molecular, Centro de Investigación y de Estudios Avanzados del Instituto Politécnico Nacional, Ciudad de México 07360, México
3 Departamento de Toxicología, Centro de Investigación y de Estudios Avanzados del, Instituto Politécnico Nacional, Ciudad de México 07360, México
4 Escuela de Ingeniería y Ciencias, Departamento de Bioingeniería, Tecnológico de Monterrey-Campus Ciudad de México, Ciudad de México 14380, México
* Correspondence: heroscar@gmail.com or ohernandez@inr.gob.mx; Tel.: +52-(55)-5999-1000 (ext. 14710)

Table S1. List of primers used in this study.

Name	Sequence 5′-3'	Annealing temperature (°C)	Product Size (bp)
NheI-ATXN7-Fw<br>*NheI-ATXN7-Rv*	CCCTCGTAAAGTCGACTATGTCGGAGCGGGCCGCGGATGA<br>CAGTTACATTGGATCCCGATCGAGTCCCTGCACGGAAACCG	66	2.679*
GAPDH Fw<br>*GAPDH Rv*	TGATGACATCAAGAAGGTGGTGAA<br>TCCTTGGAGGCCATGTGGGCCAT	64	344
TBP Fw<br>*TBP Rv*	AAAATGGTGTGCACAGGAGC<br>CTGGGTTTGATCATTCTGTAG	55	245
MBNL1 e7 Fw<br>*MBNL1 e7 Re*	GCTGCCCAATACCAGGTCAAC<br>TGGTGGGAGAAATGCTGTATGC	64	270/216
MBNL2 e7 Fw<br>*MBNL2 e7 Fw*	ACAAGTGACAACACCGTAACCG<br>TTTGGTAAAGGATGAAGAGCACC	60	266/212
APP e8 Fw<br>*APP e8 Fw*	CCACAGAGAGAACCACCAGCATTGC<br>GATACTTGTCAACGGCATCAGGGG	55	342/285
MAPT e10 Fw<br>*MAPT e10 Rv*	CTCCAAAATCAGGGGATCGC<br>CCTTGCTCAGGTCAACTGGT	58	272/197

**NheI-ATXN7* primers were used to amplify the full-length *ATXN7* carrying 10 CAG repeats (2,679 kb) to clone it into the NheI site of pTRE3G-Myc vector; *GAPDH* or *TBP* primers were used to homogenize cDNA samples in RT-PCR experiments; The rest of the primers were used to evaluate alternative splicing of the indicated exons/genes. PCR amplicon sizes observed in the presence or absence of the corresponding exons are shown.

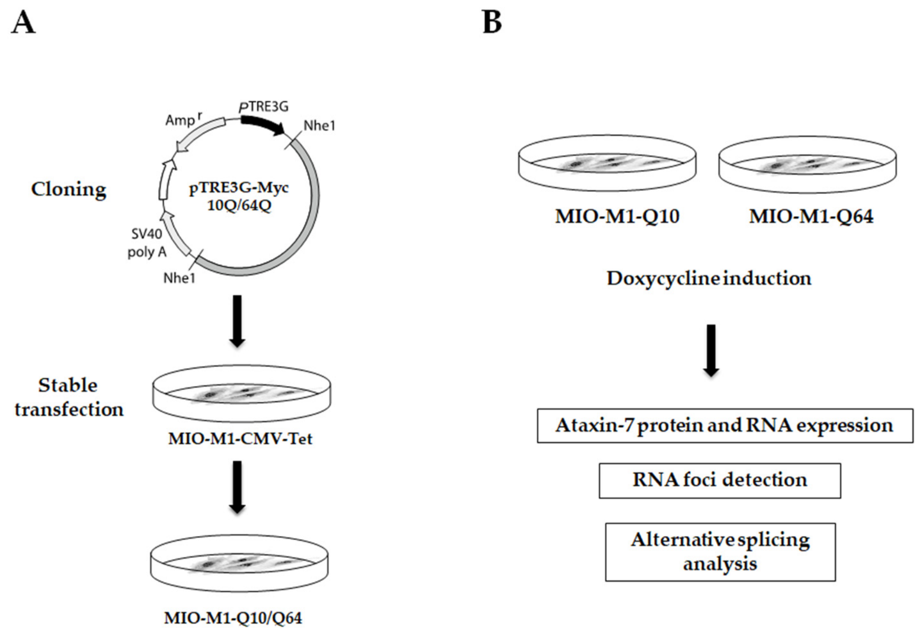

Figure S1. *General workflow. (**A**) After ATXN7 cloning, pTRE3G-Myc-10Q and pTRE3G-Myc-64Q plasmids were stably transfected on MIO-M1-CMV-Tet cells to generate MIO-M1-Q10 and MIO-M1-Q64 cells. (**B**) MIO-M1-Q10 and MIO-M1-Q64 cells were cultured in the presence or absence of doxycycline to analyze ataxin-7 protein and transcript expression, RNA foci formation by RNA FISH, and alternative splicing defects by RT-PCR.*

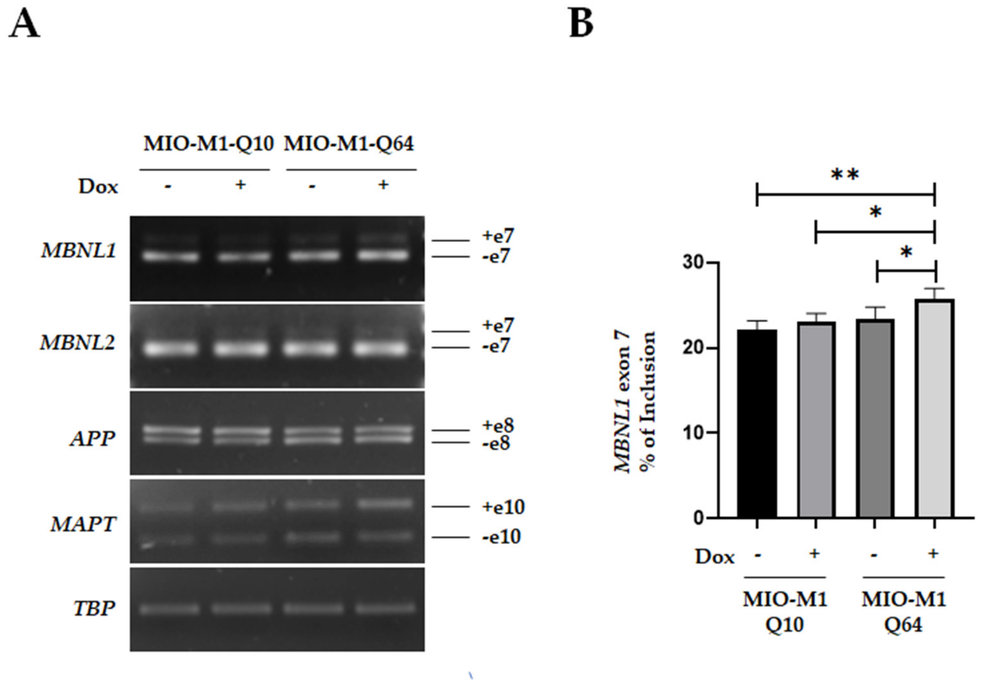

Figure S2. *Alternative splicing of exon 7 in MBNL1 is dysregulated in MIO-M1-Q64 cells. (**A**) Alternative splicing of the indicated exons was assessed by RT-PCR in MIO-M1-Q10 and MIO-M1-Q64 cells cultured in the absence (-Dox) or the presence of 1µg/mL doxycycline (+Dox) for three days. Representative images of three independent experiments are shown. TBP expression was used as endogenous control. (**B**) The percentage of splicing inclusion of MBNL1 exon 7 was calculated. Data shown are means ± SEM of independent experiments, with significant differences determined by a one-way ANOVA analysis. * and ** on graph denote $p < 0.05$ and $p < 0.005$, respectively.*
